# Covered self‐expandable metallic stent placement for tumor bleeding from duodenal invasion in patients with unresectable pancreatic cancer

**DOI:** 10.1002/deo2.361

**Published:** 2024-04-09

**Authors:** Taro Shibuki, Ko Fukushi, Kanae Inoue, Tomonao Taira, Tomoyuki Satake, Kazuo Watanabe, Mitsuhito Sasaki, Hiroshi Imaoka, Shuichi Mitsunaga, Masafumi Ikeda

**Affiliations:** ^1^ Department of Hepatobiliary and Pancreatic Oncology National Cancer Center Hospital East Chiba Japan; ^2^ Department for the Promotion of Drug and Diagnostic Development, Division of Drug and Diagnostic Development Promotion Translational Research Support Office, National Cancer Center Hospital East Chiba Japan

**Keywords:** duodenal stent, pancreatic cancer, SEMS, self‐expandable metallic stent, tumor bleeding

## Abstract

Patients with unresectable pancreatic cancer often present with duodenal bleeding, a potentially life‐threatening complication. In our case series of six unresectable pancreatic cancer patients with tumor bleeding, we explored the efficacy and safety of placement of a covered self‐expandable metallic stent in the duodenum as a treatment option; we achieved a hemostasis rate of 67% (4/6), with a rebleeding rate of 50% (2/4). No complications occurred with stent placement, except for food impaction in one patient. Covered self‐expandable metallic stent placement is a moderately effective treatment option for tumor bleeding in patients with unresectable pancreatic cancer. Although its hemostatic efficacy is limited, covered self‐expandable metallic stent placement is safe and beneficial in some cases, warranting consideration in this disease setting with limited treatment options.

## INTRODUCTION

Patients with unresectable pancreatic cancer (uPC) often experience tumor‐related complications, including anorexia, abdominal pain, jaundice, and gastrointestinal bleeding (GIB). GIB is a relatively rare, occasionally life‐threatening, encountered in approximately 1.6%–13% of patients.^1^, ^2^ GIB can be caused by a peptic ulcer, ruptured varices, tumor invasion of the gastrointestinal tract (tumor bleeding), etc., and the treatment varies depending on the cause. For tumor bleeding, palliative radiotherapy (PRT) and several endoscopic treatments, such as argon plasma coagulation, and epinephrine injection, have been attempted.^3^, ^4^ However, inconsistent effectiveness of these treatments has been reported, with initial hemostasis rates in the range of 31%–40% and re‐bleeding rates in the range of 16%–80%.^4^, ^5^, ^6^ Duodenal‐covered self‐expandable metallic stents (CSEMSs) placement has served as a useful treatment for tumor bleeding from the duodenum. However, the treatment outcomes have been reported from only a limited number of case reports of patients with a variety of cancers.^7^, ^8^, ^9^ Herein, we report a series of 6 patients with uPC with tumor bleeding treated by CSEMS placement. This study was conducted with the approval of the institutional review board of National Cancer Center Hospital East (No. 2021–467) in accordance with the principles of the Declaration of Helsinki. Approval for review of the hospital records was obtained from the Institutional Review Board of the National Cancer Center, and informed consent was obtained from all patients.

## CASE REPORT

### Patients

**FIGURE 1:**
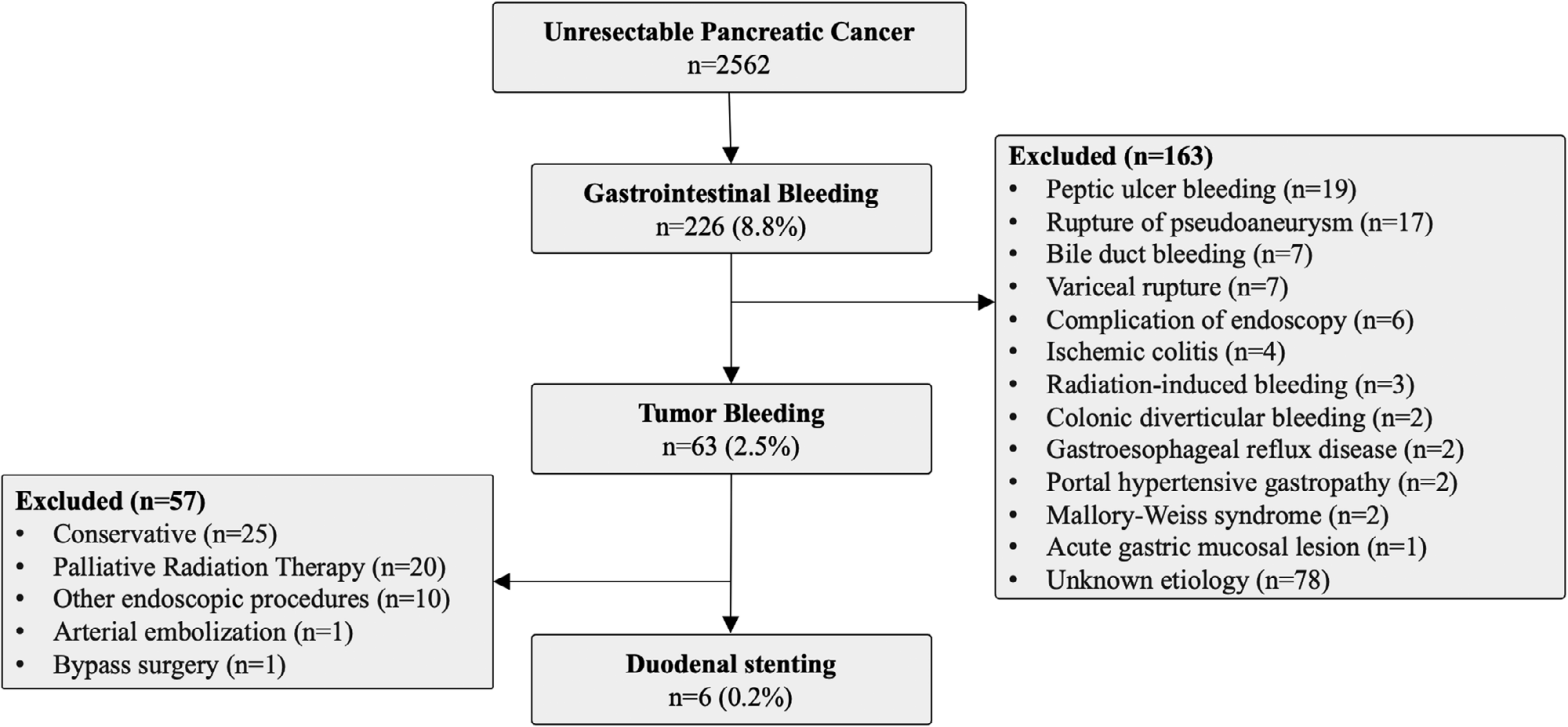
Flow diagram of the study patients.

Between May 2013 and January 2022, 2562 patients were diagnosed as having uPC at our hospital, of which 63 (2.5%) were diagnosed as having tumor bleeding. Among them, six (0.2%) were treated by CSEMS placement for tumor bleeding from duodenal invasion. Out of 63 individuals, 25 underwent conservative treatment, 20 received PRT, 10 underwent other endoscopic treatments, one underwent arterial embolization, and one underwent bypass surgery (Figure 1).

### Duodenal stenting

The GIF‐1T‐240 or TJF260V endoscope (Olympus Medical Systems) was used for all procedures. After confirming the bleeding site by endoscopy, a guidewire was inserted over the stenotic and bleeding area. Then, a contrast medium was injected to evaluate the stricture length and severity. We used the following partially CSEMS: Niti‐S D pyloric/duodenal stent (flare‐type; Taewoong Medical Co., Ltd.) measuring 60, 80, 100, or 120 mm in length and 20 mm in diameter to cover the bleeding and cancer invaded the area.

### Definition and evaluation

Endoscopic confirmation of hemostasis after placement of a CSEMS in cases of duodenal bleeding is challenging, because the mucosal/tumor surface cannot be seen directly, and endoscopic deep insertion is sometimes impossible due to severe stenosis. Therefore, we defined hemostasis as follows. The day of achievement of hemostasis was determined by reference to previous reports,^3^ as the first day after the placement of a CSEMS on which the following criteria (i)–(iii) were met consistently for at least seven consecutive days: (i) increase of the blood hemoglobin level to a value equal to or greater than that prior to the CSEMS placement; (ii) no evidence of tarry stools or hematemesis; and (iii) no need for blood transfusion. The red blood cell (RBC) transfusion volume was compared between the 4 weeks prior to the initiation of PRT and 4 weeks after the start of PRT.

The characteristics of all six patients are summarized in Table 1.

**TABLE 1:** Baseline characteristics.

No.	Age	Sex	Stage	Antithrombotic agents	Initial symptom	Bleeding site^*^	Degrees of bleeding	Duodenal stenosis	Prior USEMS placement for duodenal stenosis	Prior treatment for tumor bleeding
1	47	F	IV	−	Tarry stool	2nd portion	Oozing	Yes	No	No
2	83	M	IV	+	Hematochezia	1st–2nd portion	Oozing	Yes	No	No
3	41	M	IV	−	Tarry stool	2nd portion	Oozing	Yes	Yes	No
4	66	F	IV	−	Hematemesis	2nd portion	Oozing	Yes	Yes	No
5	50	M	IV	−	None	2nd portion	Oozing	Yes	No	No
6	67	M	IV	−	Tarry stool	2nd–3rd portion	Oozing	Yes	No	No

Abbreviations: CSEMS, covered self‐expandable metal stent; OS, overall survival; USEMS, uncovered self‐expandable metal stent.

* In all cases, the tumor bleeding was caused by duodenal invasion.

Table 2 outlines the details of the CSEMS and the treatment outcomes.

**TABLE 2:** Details of the covered self‐expandable metallic stents used, the treatment outcomes, and the clinical course.

No.	Length and diameter of the CSEMS^*^	Successful hemostasis	Complications	Rebleeding	Treatment for bleeding after CSEMS placement	Chemotherapy after CSEMS placement	OS (days)	Death
1	12 cm × 20 mm + 8 cm × 20 mm	Yes	No	No	No	No	33	+
2	12 cm × 20 mm + 6 cm × 20 mm	Yes	Food impaction	No	No	No	14	+
3	10 cm × 20 mm	Yes	No	Yes	CSEMS placement	No	50	+
4	12 cm × 20 mm	Yes	No	Yes	No	No	74	+
5	12 cm × 20 mm	No	No	−	PRT	No	44	+
6	8 cm × 20 mm	No	No	−	No	No	48	+

Abbreviations: CSEMS, covered self‐expandable metallic stent; OS, overall survival; PRT, palliative radiotherapy.

* The Niti‐S D pyloric/duodenal stents (flare‐type) were used.

**FIGURE 2:**
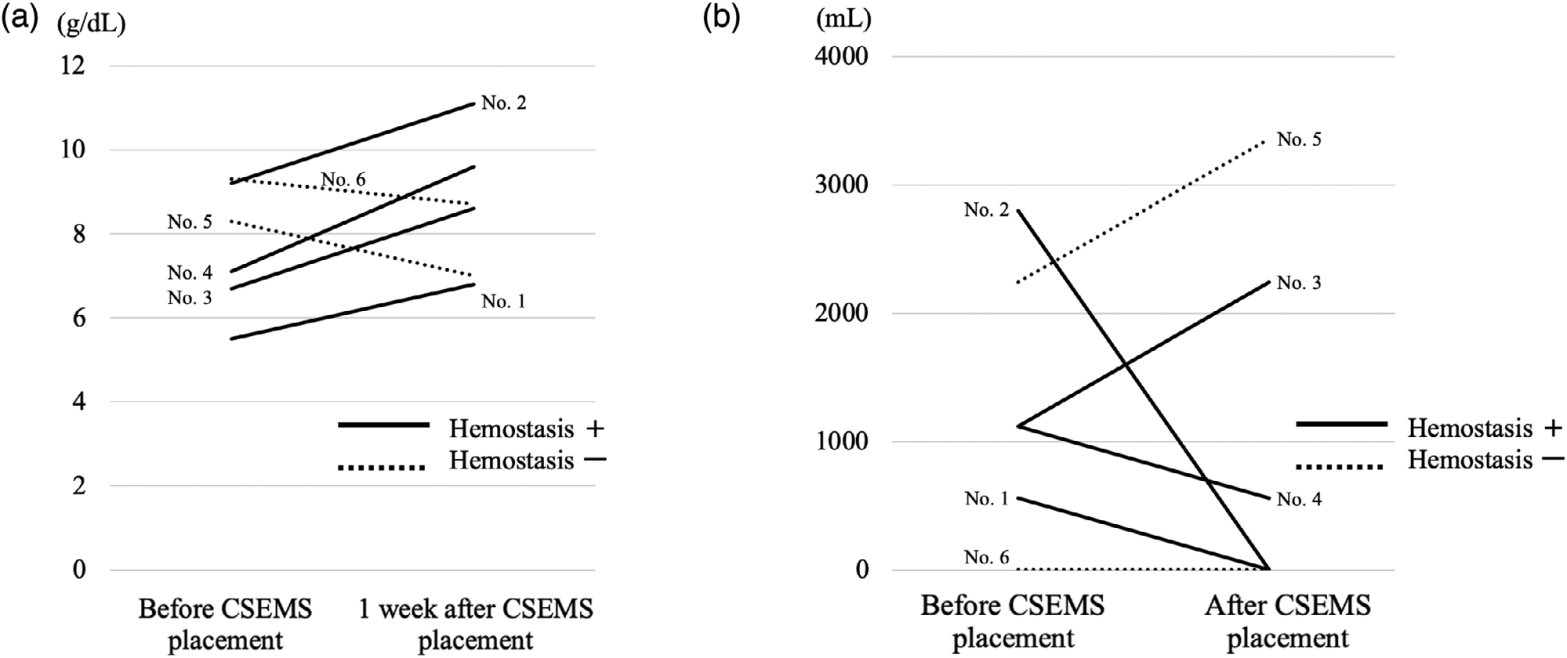
(a) Hemoglobin levels before and after placement of the partially covered self‐expandable metallic stent. (b) The volume of red blood cell transfusion before and after placement of a covered self‐expandable metallic stent. CSEMS, covered self‐expandable metallic stent.

Figure 2 summarizes the hemoglobin levels and the RBC transfusion volume pre‐ and post‐CSEMS placement.

### Case 1 (No. 1)

A 47‐year‐old woman with abdominal discomfort was diagnosed as having PC with liver metastasis. After the failure of the first‐line treatment with gemcitabine plus nab‐paclitaxel (GnP), the second‐line treatment with S‐1 was initiated. During this treatment, she developed tarry stools and anemia. Endoscopic examination revealed tumor bleeding due to extensive duodenal invasion by the PC. The length of the malignant stenosis of the duodenum was long, and we placed two CSEMSs. Hemostasis was achieved, enabling the resumption of a normal diet. No rebleeding occurred until her subsequent death.

### Case 2 (No. 4)

A 66‐year‐old woman with obstructive jaundice was diagnosed with PC with liver metastasis. After initial improvement of jaundice, she underwent first‐line GnP therapy, which had to be stopped due to severe adverse events, including fatigue and appetite loss. Second‐line treatment with S‐1 was initiated. However, the tumor progressed, leading to extensive duodenal stenosis, necessitating uncovered SEMS placement. While initially favorable, on day 13 post‐SEMS placement, the patient required emergency hospitalization for hematemesis. Endoscopic examination revealed ulcer formation and oozing at the SEMS site. Hemostasis was achieved by placing a CSEMS. Although bleeding was controlled, hematemesis recurred on day 21 post‐CSEMS placement. Due to the patient's deteriorating condition, a palliative care approach was adopted.

### Case 3 (No. 5)

A 50‐year‐old man with abdominal pain was diagnosed as having PC with liver metastasis. Tumor bleeding was incidentally observed during endoscopic biliary drainage. Despite conservative management, the anemia persisted. A CSEMS was placed to cover the tumor, securing the proximal end of the stent to the gastrointestinal tract with clips to prevent dislocation. However, the anemia continued to worsen slowly, necessitating periodic blood transfusions. On day 10 post‐CSEMS placement, we initiated the patient on PRT for tumor bleeding at the dose of 3 Gy administered in 10 fractions, achieving hemostasis. However, due to disease progression and declining condition, chemotherapy wasn't feasible, and palliative care was initiated.

## DISCUSSION

We report the largest case series of uPC patients treated by duodenal CSEMS placement for tumor bleeding. Our study demonstrated CSEMS placement as a moderately effective and safe treatment method, achieving hemostasis in 67% (4/6) with a rebleeding rate of 50% (2/4). No severe complications occurred. These findings suggest that CSEMS placement is a feasible treatment option for tumor bleeding from duodenal invasion.

If GIB is suspected, we perform contrast‐enhanced CT and endoscopy to locate the bleeding site. For arterial bleeding, embolization is performed. In cases of venous bleeding, conservative treatment is initiated, with PRT administered if hemostasis isn't achieved. Duodenal stenting is considered if there's duodenal stenosis with a low risk of stent migration. Endoscopic CSEMS placement is an effective treatment for esophageal variceal bleeding, with a reported clinical success rate of 96% according to a meta‐analysis covering 155 patients with esophageal variceal bleeding.^10^ However, it has rarely been implemented for tumor bleeding, with only a few case reports published to date. Yamashita et al. reported that deployment of a duodenal CSEMS is a useful salvage therapy for bleeding caused by advanced pancreatobiliary cancer, with a hemostasis rate of 100%.^8^ Other than that, only a few successful cases in patients with duodenal cancer and hepatocellular carcinoma have been reported.^9^ In our study, hemostasis was achieved in four out of the six patients (67%). However, comparison with previous reports might not be reasonable, because of the different hemostasis definitions and patient conditions. The most reliable method for assessing hemostasis is direct observation through endoscopy. However, after the placement of a duodenal stent, it is often challenging to evaluate hemostasis by endoscopy because the mucosal/tumor surface is covered by the covered stent and the stenosis makes deeper insertion of the scope difficult. Therefore, our study defined hemostasis based on objective measurements referencing previous studies.^3^

In patients achieving hemostasis with CSEMS placement, we observed improved anemia and reduced RBC transfusion requirements. Although subsequent initiation of chemotherapy wasn't always possible, the impact of CSEMS placement on the quality of life makes it worthy of further investigation. Among the four patients in whom hemostasis was achieved, two developed rebleeding, contrasting previous studies with no rebleeding reports.^8^, ^9^ Both patients with rebleeding were undergoing uncovered SEMS placement for severe duodenal stenosis, suggesting a higher risk of rebleeding. Rebleeding can be addressed by PRT, as demonstrated in No. 5 and reported in a previous study.^3^ This minimally invasive approach is worth considering in this setting, where treatment options are limited. Complications associated with CSEMS placement, except food impaction in one patient, were absent, consistent with prior reports.^8^, ^9^ In conclusion, CSEMS placement is moderately useful for tumor bleeding in patients with uPC. Although its sustained hemostatic efficacy was limited, it proved safe and was beneficial in some cases, making it worthy of consideration in this setting with limited treatment options.

## CONFLICT OF INTEREST STATEMENT

Masafumi Ikeda has received research funding (institution) from AstraZeneca, Bayer, Bristol‐Myers Squibb, Boehringer Ingelheim, Chugai, Chiome Bioscience, Delta‐Fly Pharma, Eisai, Eli Lilly Japan, Invitae, MSD, J‐Pharma, Merck biopharma, Merus N.V., Novartis, Nihon Servier, Ono, Pfizer, and Syneos Health, has consulted for AstraZeneca, Chugai, MSD, Nihon Servier, and Novartis, and received speaker honoraria from AbbVie, AstraZeneca, Chugai, Eisai, Eli Lilly Japan, Fujifilm Toyama Chemical, Guardant Health Japan, Incyte Biosciences Japan, MSD, Nihon Servier, Novartis, Nippon Kayaku, Ono, Taisho Pharmaceutical, Teijin, Takeda, Taiho, and Yakult.

Shuichi Mitsunaga has received research funding from Chugai, Astellas, Toray, Ajinomoto, and Pfizer, and received speaker honoraria from Ono, Toray, and Otsuka.

Hiroshi Imaoka has received research funding from Ono Pharmaceutical and Nihon Servier, is a member of the advisory board for Nihon Servier, received speaker honoraria from Yakult, Nihon Servier, Kaneka Medix, Boston Scientific, Medico's Hirata, and SB‐Kawasumi Laboratories, and received payment for expert testimony from Kaneka Medix.

The other authors declare no conflict of interest.

## PATIENT CONSENT STATEMENT

Informed consent was obtained from all patients.

## Data Availability

We would like to provide data as needed.
